# Calculous Diseases in the Mauritius

**Published:** 1844-01

**Authors:** Robert Allen

**Affiliations:** Staff Surgeon 2d class H.M. service


### CALCULOUS DISEASES IN THE MAURITIUS.

Mauritius; 22d July, 1843.

*To the Editor of the British and Foreign Medical Review.*

SIR,—In the number of your Review for April, 1843 (which reached this a few days ago), I saw in the criticism of Rayer on the 'Disease of the Kidney' the following sentence: "The frequency of this gravel (lithic acid) in the youth of the Mauritius makes it a question of interest whether adults suffer in a higher proportion from stone than those in other countries: this M. Rayer's information does not enable him to say." (p. 482.)

As I have been, through the kindness of the medical practitioners, made acquainted with most cases of importance in surgery which have occurred since my arrival here in 1832, I think myself competent to give the information you are anxious to obtain; and, since reading the number of your Review referred to, have taken the precaution of consulting most of the practitioners of the island on this point.

During the last ten years, out of an average population of 111,731 (from official documents), I am only aware of twelve cases of stone in the bladder, one of which occurred in a female: of course those in which small calculi have been voided by the efforts of the patient's bladder are not included. Of the calculi in the twelve cases alluded to, five were broken up with the lithontriptor, by Mr. Rogers and Dr Dunbar, (two successfully, and three died from inflammation of the bladder, or constitutional irritation.) The sixth was that of an Irish lad aged 16, six years in the colony, who died at the Civil Hospital, in whom a large calculus of triple phosphate was found after death. In the seventh, a stone the size of an almond was propelled into the membranous part of the urethra, and was cut down on by Dr. Penison of the 87th regiment. It slipped back into the bladder, but some weeks later was expelled by the artificial opening in the perineum, and is now in my possession. It is composed of phosphate of lime. The eighth was that of a boy eight years of age, from whom I extracted a calculus, of two inches long by one in diameter (weighing ten drachms, sixteen grains), by the lateral operation performed with Mr. Liston's knife. This calculus was analysed by my friend Mr. Rogers, who found that it was composed of ammoniaco-magnesian phosphate, with a little phosphate of lime and small quantities of lithate of lime and ammonia, on a nucleus of lithic acid. The patient walked out on the twenty-first day after the operation, and soon regained excellent health. The three remaining male cases were lost sight of. In the female a calculus the size of an almond, being entirely composed of phosphate of lime, projected from the meatus urinarius while voiding urine, and she herself pulled it out.

Discharges of sabulous matter with the urine are common at all ages here; and the many we have examined by the microscope were composed of lithic acid, with the exception of those from a native of Norfolk, England, which were crystals of triple phosphate, voided with great pain. In pigs, however, calculi of an inch or two in diameter, have been frequently found in the urinary bladder after death, generally composed of carbonate of lime: in the centre of one we observed a piece of straw two lines in length. Your obedient servant,

ROBERT ALLEN, Staff Surgeon 2d class H.M. service.

